# Mechanical Performance of Warm-Mixed Porous Asphalt Mixture with Steel Slag and Crumb-Rubber–SBS Modified Bitumen for Seasonal Frozen Regions

**DOI:** 10.3390/ma12060857

**Published:** 2019-03-14

**Authors:** Yongchun Cheng, Chao Chai, Chunyu Liang, Yu Chen

**Affiliations:** College of Transportation, Jilin University, Changchun 130025, China; chengyc@jlu.edu.cn (Y.C.); chaichao18@mails.jlu.edu.cn (C.C.); chenyu17@mails.jlu.edu.cn (Y.C.)

**Keywords:** porous asphalt mixture, crumb-rubber–SBS modified bitumen, warm mix asphalt, steel slag, mechanical performance

## Abstract

In this paper, the performance of a warm-mixed porous asphalt mixture (PAM) with steel slag as aggregate and crumb-rubber–SBS (styrene-butadiene-styrene) modified bitumen as a binder was studied. Two kinds of warming additives were used, namely ethylene bis stearic acid amide (EBS) and stearic acid amide (SA). The mixtures were investigated for their permeability, Marshall stability, low-temperature crack resistance, and underwent a rutting test, water sensitivity evaluation and Cantabro particle loss test. Then, the viscoelastic and dynamic characteristics of the mixtures were also analyzed. The results showed that the addition of the warming additives allowed the decrease of the manufacturing temperature by 10 °C. Thus, the addition of warming additives significantly improves the low-temperature crack resistance and slightly reduces the water sensitivity, weakly increases the permeability, and has little effect on the resilient modulus. Since the addition of SA significantly improves the low-temperature crack resistance and rutting resistance of the PAM, SA is therefore recommended for pavement engineering in seasonal frozen regions.

## 1. Introduction

With the development of the transportation industry, the pavement structure is required to have higher service performance, and so porous asphalt mixture (PAM) has been rapidly developed. This mixture has large porosity, and rainwater can be discharged rapidly through vertical seepage. Therefore, the friction between the tires and the road surface is effectively guaranteed, which greatly improves the safety of driving on rainy days [1,2,3]. In addition, the PAM has good sound absorption and noise reduction performance, and studies have shown that the noise reduction of the pavement can reach 3-5 Db [4,5]. Thus, PAM has been widely studied and applied in many countries. However, a high mixing temperature is required due to the high-viscosity bitumen used in PAM; therefore, PAM is likely to cool down during the paving process, resulting in insufficient compaction and early damage to the road surface, which brings difficulties for paving in seasonal frozen regions.

Warm-mix asphalt can not only reduce the production temperature of the mixture, decrease energy consumption and harmful gas emissions, but also does not cause an apparent weakening of pavement performance [6,7]. Furthermore, it can be achieved by using chemical additives without implementing important changes in equipment [8]. Many scholars have done a great deal of research on Sasobit as a warming additive [9,10,11,12,13], and have uncovered some useful research results. However, there are some disadvantages to using Sasobit as a warming agent. First, it will weaken the low-temperature crack resistance of PAM, which will cause the premature failure of the mixture in cold regions [14,15]. Moreover, the application of Sasobit greatly increases the cost of the mixture. Besides, there is almost no research on the use of ethylene stearic acid amide (EBS) and stearic acid amide (SA) as warming agents. The cost of these two substances (EBS and SA) is only 47.5% and 62.5% of the cost of Sasobit in China.

In addition, the application of construction waste such as steel slag and recycled aggregate has been extensively studied in recent years [16,17]. Liu et al [18] studied the use of steel slag instead of a part of the aggregate, while the incorporation of steel fibers can improve the mechanical properties and thermal conductivity of the mixture; however, the case where the natural aggregate is completely replaced by steel slag was not considered. Der-Hsien Shen [19] pointed out that PAM made from steel slag as an aggregate has better slip resistance and rutting resistance, but it has not been analyzed for its low-temperature crack resistance. Wu et al. [20] found that using steel slag powder as a filler in hot-mix asphalt mixtures could replace mineral filler in asphalt concrete (AC) with better pavement performance. A study [21] has shown that adding slag to the mixture can significantly increase its fatigue life, and the fatigue life increases as the slag content increases. Martinho [22] investigated the mechanical performance of warm-mix asphalt blends with electric arc furnace slag (EAFS) as a substitute of part of the aggregate and showed that the introduction of EAFS into the warm-mix asphalt blends increases the Marshall stability but slightly decreases water sensitivity. Tam [23] studied the applicability of steel slag in asphalt mixtures for self-healing purposes and showed that the substitution of 30% of normal coarse aggregate by steel slag is very promising, because its presence not only provides better healing results but also helps the whole mixture to improve its load–displacement relationship with higher ductile behavior.

In addition, the utilization of crumb-rubber in the asphalt mixture can improve its mechanical properties [24,25]. C. Sangiorgi [26] studied the effectiveness of adding crumb-rubber to PAMs and showed that the application of crumb-rubber improves the bitumen/aggregate affinity and decreases susceptibility to thermal cracking. He also proposed a new dry-hybrid technology to use crumb-rubber for the production of stone matrix asphalt (SMA), and results showed that it seems to be a viable solution for the production of eco-friendly mixtures with performances comparable to traditional SMA [27]. Chen et al. [28] proposed a new treatment method of crumb-rubber and showed that the high-temperature rheological properties of rubberized binders were improved, but it is only recommended to be applied on typical rubber to obtain promotions towards rheological performances at high temperatures. C. Loderer et al. [29] found that the addition of crumb-rubber to bitumen increases the complex modulus at high temperatures and reduces stiffness at low temperatures. He et al. [30] investigated the temperature sensitivity characteristics of bitumen after different aging processes and showed that SBS/CRP(crumb rubber powder)-modified bitumen has a strong anti-aging ability in that its flexibility and structure remain in good condition after long-term aging.

In this paper, firstly, three sets of PAM with steel slag as aggregate and crumb-rubber–SBS modified bitumen as a binder were designed and produced. The first group was not supplemented with a warming additive as a reference. The other two groups were supplemented with different warming additives, namely ethylene bis stearic acid amide (EBS) and stearic acid amide (SA). Then, the mechanical properties of PAMs were analyzed through experiments. Finally, whether the warm PAMs can be applied to pavement engineering in seasonal frozen areas was discussed.

## 2. Materials and Methods 

### 2.1. Raw Materials

#### 2.1.1. SBS-Modified Bitumen

The SBS-modified bitumen used in this study was supplied by Xinda company in Liaoning Province, China. Its main properties are listed in Table 1.

#### 2.1.2. Crumb-Rubber and Warming Additives

The crumb-rubber used in this research is 40 mesh, which was produced from a chemical plant (Hongda, Jilin, China). Referring to the existing research [31], the amount of crumb-rubber used in this study was 10% of the mass of SBS-bitumen. Its main properties are presented in Table 2. 

**Figure 1:**
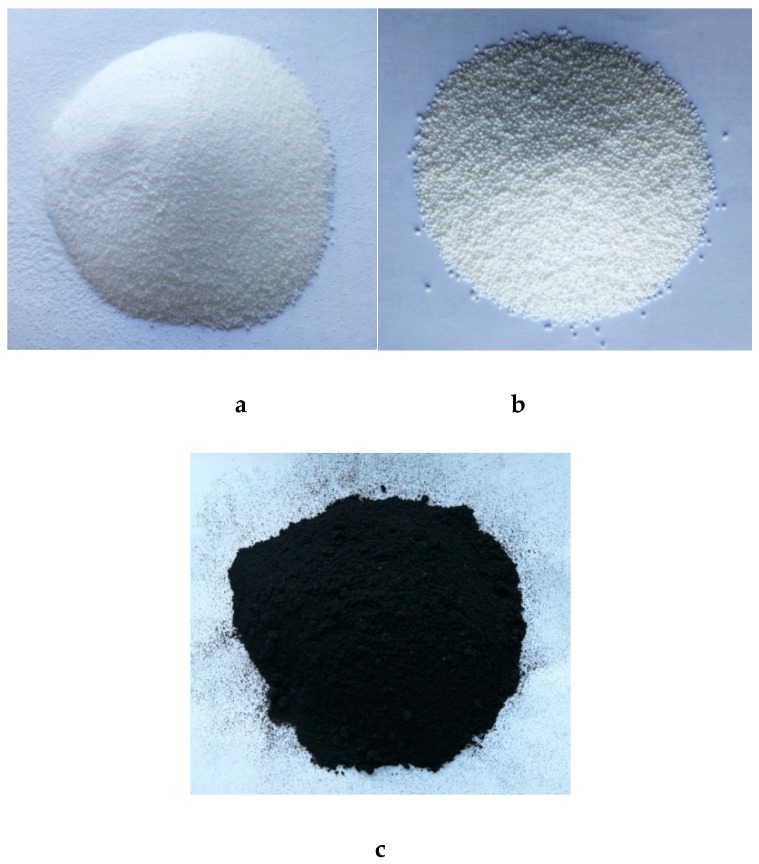
(**a**) Ethylene bis stearic acid amide (EBS); (**b**) stearic acid amide (SA); (**c**) crumb-rubber.

There are two kinds of warming additives used in this study, namely ethylene bis stearic acid amide (EBS) and stearic acid amide (SA), both of which were produced in a chemical company (Tianyu, Sichuan, China). The amount of the two warming additives in the mixture was 3% of the mass of the asphalt. The appearance of the rubber powder and additives is shown in Figure 1.

In order to uniformly disperse crumb-rubber and warming additives in SBS-modified bitumen, firstly, 700 g of SBS-modified bitumen was put into the beaker, and then the beaker was stored in an oil bath at 170 °C for 30 min. Next, we added the appropriate mass fraction of warming additives and rubber powder, using a high-shear homogenizer to blend at a speed of 4000 rpm for 40 min. Finally, we heated them in the oil bath at 170 °C for 30 min. For the crumb-rubber SBS-modified bitumen without the warming additives, the remaining steps are the same as above, except that the warming additives were not added.

#### 2.1.3. Aggregate

The aggregate used in this research is the steel slag produced by Jilin Dongsheng company (Jilin, China). In order to avoid the problem of the volume expansion of steel slag, it was placed in the natural environment for 3 years. Like the natural aggregate, its particle size distribution ranges from 0.075 mm to 13.2 mm. It could be applied to the experiment after simple screening. The physical properties of steel slag were determined according to the Test Methods of Aggregate for Highway Engineering (JTG E42-2005) [32]. Its main properties are presented in Table 3.

#### 2.1.4. Design and Production of PA Mixture

**Figure 2:**
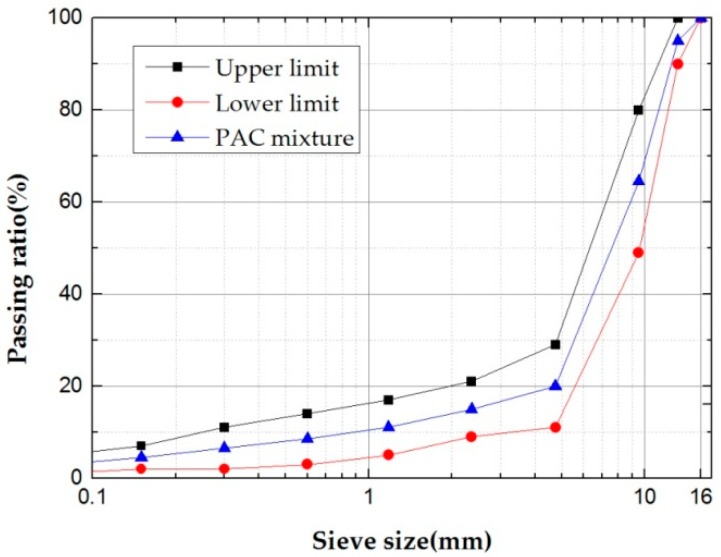
Curves of PAC mixture.

First, the gradation was determined according to the Technical Specification for Permeable Asphalt Pavement (CJJ/T 190-2012) [33], and the gradation curve is shown in Figure 2. Then, standard Marshall specimens were made, according to the Standard Test Methods of Bitumen and Bituminous Mixtures for Highway Engineering (JTG E20-2011) [34]. First, we put the weighed steel slag into a constant temperature mixing pot and stirred it for 90 s, then we added the corresponding quality of crumb-rubber–SBS modified bitumen and stirred for 90 s. Finally, the corresponding quality of mineral filler is added, and the mixture is stirred for 90 s to prepare the specimens. The asphalt–aggregate ratio of all the specimens is 4.0. 

The first group of specimens was not supplemented with warming additives and was set as a control group. The remaining two groups of specimens were supplemented with EBS and SA, respectively, represented by EBS-PAM and SA-PAM. All parameters of the PAM were tested with three specimens in each group, and the test results are expressed by the mean. All specimens were compacted with a standard Marshall compacting hammer 50 times per side, and the height of the specimens as in the range of 63.5 ± 1.3 mm.

### 2.2. Test Methods

#### 2.2.1. Viscosity

A Brookfield viscometer (KRH-I, Shanghai Konmix Mechanical & Electrical Equipment Technology Co. Ltd., Shanghai, China) was used to evaluate the viscosity of three types of modified bitumen according to Chinese standards GB/T 0625-2011. The test temperature as from 100 to 190 °C, at 10 °C intervals. 

#### 2.2.2. Void Characteristics and Permeability

**Figure 3:**
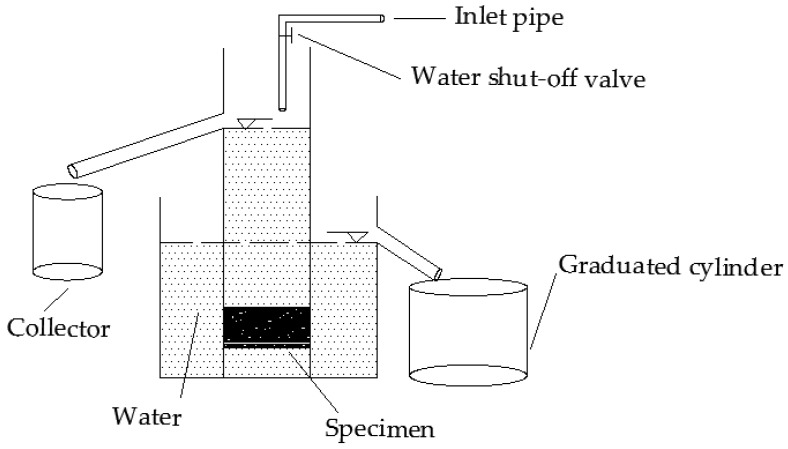
Schematic diagram of apparatus.

The voids in the mixture are important indicators in PAM which directly affect the permeability coefficient. First, the void characteristics of the three mixtures were tested according to the Chinese standard (T 0706-2011). Then, the permeability coefficient of the three mixtures was measured by the constant head permeameter. The hydraulic height of the device is 15 cm and the schematic diagram of the apparatus is shown in Figure 3. During the experiment, the water flow switch was turned on, and the water flow speed was adjusted until the upper and lower overflow pipes had a stable water flow, and the water flowing out of the overflow tank was taken up with a water tank of 5000 mL. We recorded the time it takes to collect water from the beginning to collect 5000 mL of water. Research [35] has shown that when the test was performed with a low hydraulic gradient, the constant head permeability test followed Darcy’s law. Therefore, based on Darcy’s law, the calculation formula of the water permeability coefficient can be derived according to Equation (1).

(1) $$K=\frac{QL}{At\Delta h}$$

where *K* is the permeability coefficient (cm/s); *Q* is the amount of the water permeating through the specimen (5000 mL); *L* is infiltration length (cm); *A* is cross-sectional area of the specimen (cm^2^); *t* is the time from the start of water collection to the filling of 5000 mL of water; and Δ*h* is the water head difference (15 cm).

#### 2.2.3. Marshall Test

The Marshall stability test was carried out on three kinds of PAMs according to the Chinese standard (GB/T 0709-2011). Before the experiment, the specimen was placed in a constant-temperature water bath at 60 °C for 30 min, and the upper and lower indenters of the Marshall tester were also placed in a constant-temperature water bath at 60 °C, and returned to the original position of the tester after reaching that temperature. Then, we placed the specimen between the upper and lower indenters. The Marshall tester was started and the Marshall stability and flow value were recorded after the end of the experiment.

Moreover, in order to further analyze the strength of the mixture with steel slag as aggregate, the specimens in this study were compared with the specimens with basalt as aggregate in the existing research [36]. Except for aggregates, the other fabrication steps of the specimens in the citation are the same as those for the specimens in this study.

#### 2.2.4. Freeze–Thaw Splitting Test

According to the Chinese standard (T 0729-2000), the Marshall specimens were subjected to a freeze–thaw splitting test in order to analyze the effect of the application of the warming additives on the water stability of the PAMs. For the freeze–thaw splitting test, firstly, the specimen was kept at a vacuum of 97.3–98.7 KPa in water for 15 min, and then returned to normal pressure in water for 30 min. Then, the specimen was placed in a plastic bag, and about 10 mL of water was added to the plastic bag. After closing the plastic bag, we placed it in a refrigerator at −18 °C for 16 h. Then, we removed the specimen from the refrigerator, took the plastic bag, and immediately placed the specimen in a constant-temperature water bath at 60 °C for 24 h. Finally, the specimen was placed on the experimental machine and tested at a loading rate of 50 mm/min to obtain the maximum load.

#### 2.2.5. Low-Temperature Splitting Test

**Figure 4:**
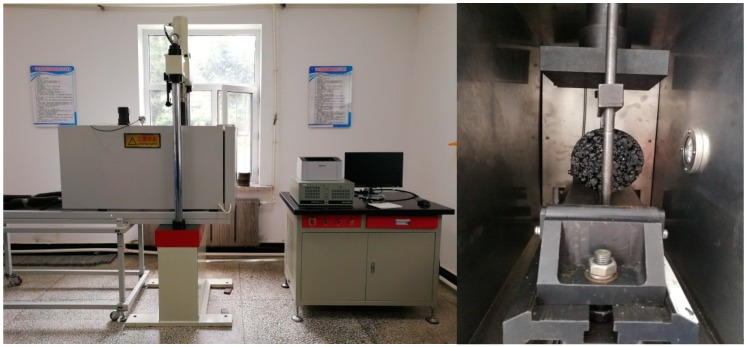
The low-temperature splitting test.

PAM must have sufficient crack resistance under low-temperature conditions, especially in seasonal frozen areas; for example, in Changchun City, there are 4 months when the ambient temperature is lower than 0 °C in one year. Therefore, asphalt pavements located in this area must have sufficient low-temperature crack resistance. In order to explore the effect of the addition of warming additives on the low-temperature performance of PAM, the low-temperature splitting test was performed according to the Chinese standard (T 0716-2011). The experimental site is shown in Figure 4. The experiment was carried out using an electro-hydraulic servo material testing machine. The range is 100 KN and the accuracy is 0.01 KN. Moreover, the testing machine has a displacement sensor which can measure the vertical deformation of the specimen. The experimental temperature is −10 °C, and the load application rate is 1 mm/min. Before the experiment, the specimens were stored for 6 h in a chamber at −10 °C.

#### 2.2.6. Rutting Test

A rutting test was used to evaluate the high temperature stability of the mixtures according to JTG E20-2011 (T 0719). The slab specimens were prepared from three kinds of PAMs, and the size was 300 mm × 300 mm × 50 mm. The specimens were tested under 0.7 MPa of repeated wheel loading at 60 °C. The wheel speed was set at 42 passes per minute. The test duration of each test piece was 60 min. During the experiment, the displacement at the test piece was recorded by the displacement sensor (Meiyu, Shanghai, China). The formula for calculating the dynamic stability is shown in Equation (2).

(2) $$DS=\frac{(t_{2}-t_{1})\times N}{d_{2}-d_{1}}\times C_{1}\times C_{2}$$

where *DS* is the dynamic stability of PAM (wheel pass/mm); *N* is wheel speed (42 times/min); *d*_1_, *d*_2_ are vertical deformations corresponding to *t*_1_, *t*_2_, (mm), and *t*_1_, *t*_2_ are the test times of 45 and 60 min, respectively; and *C*_1_ and *C*_2_ are the test machine type coefficient and the test piece coefficient, respectively, which are taken as 1.0.

#### 2.2.7. Cantabro Particle Loss Test

In order to evaluate the adhesion of aggregates to the asphalt of PAMs in this study, the Cantabro particle loss test was carried out on the three mixtures according to the Chinese standard (T 0733-2011). First, the specimen was placed in a constant-temperature water bath at 20 °C ± 0.5 °C for 20 h. Then, the specimen was taken out, its surface water wiped off and its quality recorded as *m*_0_. Finally, the specimen was quickly placed in the Los Angeles test machine (Qingda, Tianjin, China), the lid closed and the test machine switched on to at rotate 300 rpm at 30–33 r/min. After the operation of the machine was finished, the residual mass of the test piece was weighed and recorded as *m*_1_. The Cantabro particle loss was calculated according to Equation (3).

(3) $$\Delta S=\frac{m_{0}-m_{1}}{m_{0}}\times 100$$

#### 2.2.8. Creep Test

**Figure 5:**
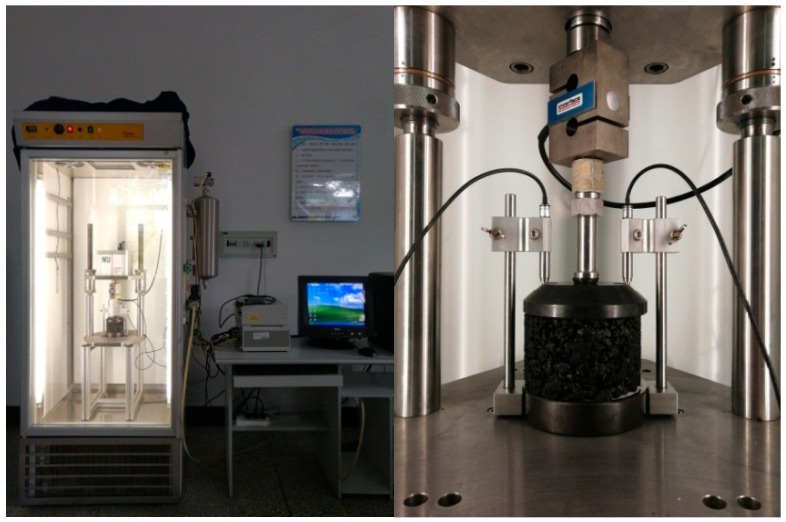
The creep test.

In order to analyze the effect of the addition of warming additives on the viscoelasticity of the mixture, the uniaxial compression static creep test was carried out on standard Marshall specimens made from three PAMs using a servo-pneumatic test machine (NU-14, Cooper Technologies Ltd, Ripley, UK). The experimental temperature was determined to be 50 °C, and the stress level was 400 KPa. Before the test, the specimen was stored for 5 hours in the machine chamber at 50 °C to ensure uniform temperature throughout the test phase. In addition, a preload of 10 KPa was applied to the specimen and held for 30 s to bring the indenter into close contact with the specimen. The experimental time was 3600 s. The experimental site is shown in Figure 5.

#### 2.2.9. Indirect Tensile Test

**Figure 6:**
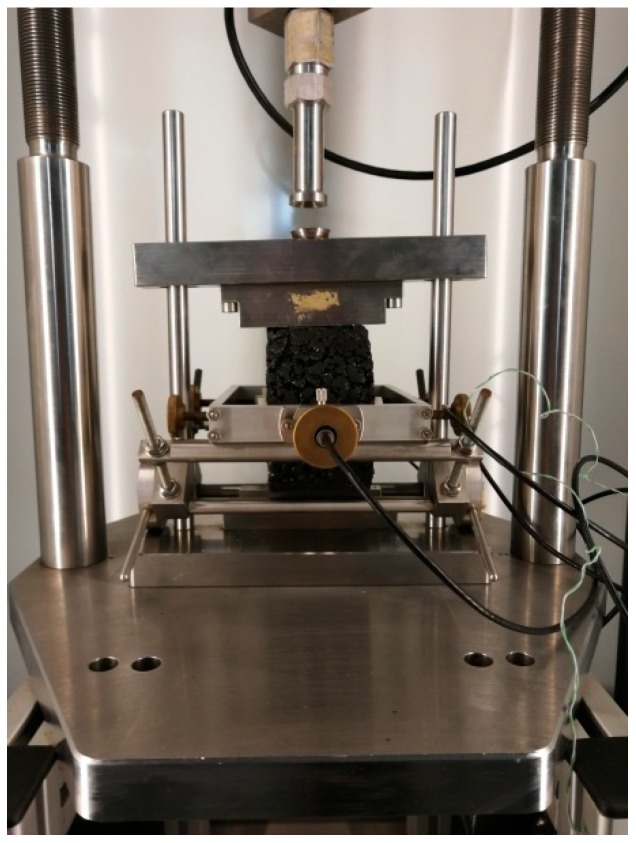
Indirect tensile test.

In order to analyze the response of PAM to traffic loading under the addition of two warming additives, the indirect tensile test was performed in accordance with BS EN 12697-26:2004, and its experimental site is shown in Figure 6. The specimens used in this study were standard Marshall specimens and were kept in the machine chamber for 5 hours at 20 °C before the experiment. The test was conducted at a temperature of 20 °C by applying compressive loads with a haversine waveform, and the loading frequency was 2 Hz.

## 3. Results and Discussion

### 3.1. Viscosity

**Figure 7:**
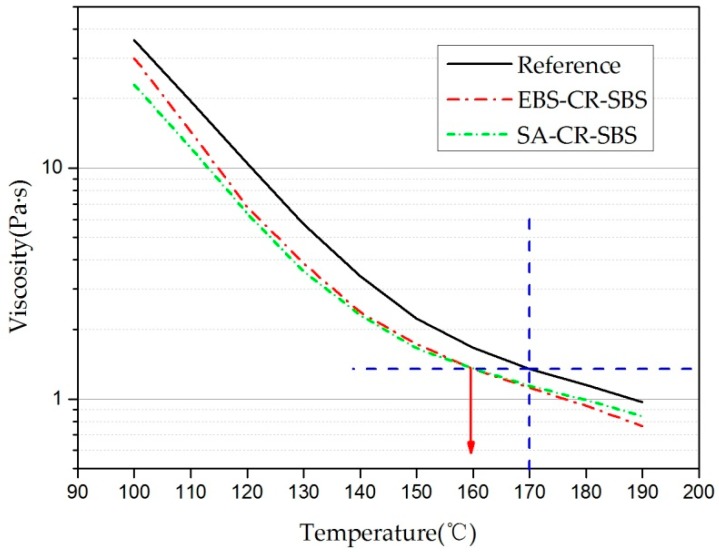
Result of the viscosity test.

As can be observed in Figure 7, the viscosity of EBS-CR-SBS and SA-CR-SBS was somewhat weakened compared to the reference. This is because EBS and SA act as synthetic waxes, which have lubricity and reduce the viscosity of the bitumen. In addition, it can be seen that, below 140 °C, the viscosity reduction effect of SA is better than that of EBS, and above 140 °C, the viscosity reduction effects of SA and EBS is basically the same. This is because the melting point of SA is approximately 100 °C, which is lower than the melting point of EBS (140 °C). Therefore, in the range of 100–140 °C, SA exhibits a more pronounced effect of reducing viscosity.

Referring to the existing research [37], the mixing temperature of the control mixture in this experiment was set to 170 °C. For the other two mixtures supplemented with the warming additives, the mixing temperature was set to 160 °C based on the principle that the viscosity of the three binders was equal when mixing.

### 3.2. Void Characteristics and Permeability

The experimental results with standard deviation (Sd) are listed in Table 4. The permeability of the three mixtures meets the requirements of the Chinese standard. In addition, the void ratios of EBS-PAM and SA-PAM increased slightly compared with the control group. This indicates that although the mixing temperature of the three mixtures is the same, the application of the warming agents still has a slight effect on the compaction of the mixture and results in a slight increase in the void ratio of the mixture, which results in the increased permeability of the two warm PAMs.

### 3.3. Marshall Stability

According to Table 5, the Marshall stability and flow value of the three mixtures meet the Chinese specifications. In particular, the Marshall stability of SA-PAM is 18.4% higher than that of the control group. This shows that the application of two kinds of warming agents not only does not reduce the Marshall stability of the mixture, but elicits a certain improvement. This may be because the warming agents have lubricity for the rubber powder, so that the rubber powder is more uniformly dispersed in the SBS-modified bitumen, and the dispersion system of the crumb-rubber–SBS-modified bitumen is more uniform, thereby improving the Marshall stability of the mixture.

As can be seen from Table 6, the specimen made of steel slag in this experiment has a superior Marshall stability to that of the specimens made of basalt. The strength of the mixture is highly dependent on the bond strength of the aggregate to the binder. The surface of the steel slag is rougher than that of the natural aggregate, so the steel slag can be better bonded to the binder. Therefore, the application of steel slag can not only realize waste utilization, but also has higher strength.

### 3.4. Water Sensitivity

It can be seen from Table 7 that the freeze–thaw splitting strength ratios of EBS-PAM and SA-PAM are reduced. That is, the addition of the warming additives reduces the water stability of the warm-mixed PAMs, but they still meet the requirements of Chinese regulations. Furthermore, the increase in the air void content reduces the water stability of the mixture, which is consistent with Maria Rodriguez-Alloza’s research [38].

### 3.5. Low-Temperature Mechanical Performance

**Figure 8:**
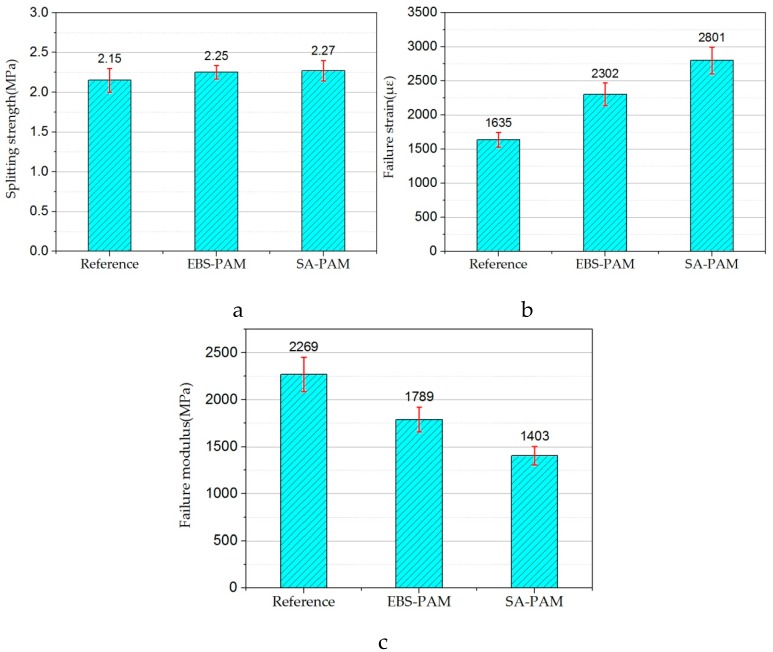
(**a**) The splitting strength; (**b**) the failure strain; (**c**) the failure stiffness modulus.

It can be seen from Figure 8 that the incorporation of EBS and SA effectively improves the low-temperature performance of the mixture. Specifically, Figure 8a indicates that the splitting strength of EBS-PAM and SA-PAM increased by 4.7% and 5.5%, Figure 8b shows that the failure strain of EBS-PAC and SA-PAC increased by 41% and 71%, and in Figure 8c, it can be seen that the failure stiffness modulus decreased by 21% and 38%, respectively, compared with the control group. 

As a conclusion, the incorporation of EBS and SA improves the low-temperature deformability of the mixture, reduces the failure stiffness modulus and improves the low-temperature crack resistance of the mixture. This may be because the application of the warming agents makes the crumb-rubber distribution in the binder more uniform, and the crumb-rubber in the modified bitumen could increase the toughness of the binder, thereby improving the low-temperature crack resistance of the mixture. The improvement effect of SA is more remarkable. Therefore, SA is more suitable for seasonal frozen areas.

### 3.6. High-Temperature Mechanical Performance

It can be seen from Table 8 that the type of warming agent has a great influence on the high-temperature stability of the PAM in this study. For example, the dynamic stability of EBS-PAM was decreased by 59.5% compared to the control group, while the dynamic stability of SA-PAM was increased by 25% relative to the control group, which shows that the application of EBS greatly weakens the high-temperature stability of the PAM; in contrast, the application of SA has a significant improvement on the high-temperature stability of the PAM.

According to the Technical Specification for Permeable Asphalt Pavement (CJJ/T 190-2012), the dynamic stability of PAM must be greater than 3500 wheel pass/mm Therefore, SA-PAM is more suitable for pavement engineering because of its outstanding rutting resistance.

### 3.7. Cantabro Particle Loss Test

From the results in Table 9, the Cantabro particle loss of EBS-PAM and SA-PAM increased by 4.3% and 2.8%, respectively, relative to the control group. This indicates a certain weakening of the adhesion between the aggregate and the binder in the warm-mix PAMs. Nevertheless, according to the Technical Specification for Permeable Asphalt Pavement (CJJ/T 190-2012), the Cantabro particle loss of the PAMs shall not exceed 15%. Therefore, the Cantabro particle loss of the three PAMs in this experiment meets the specification requirements.

### 3.8. Viscoelastic Properties 

**Figure 9:**
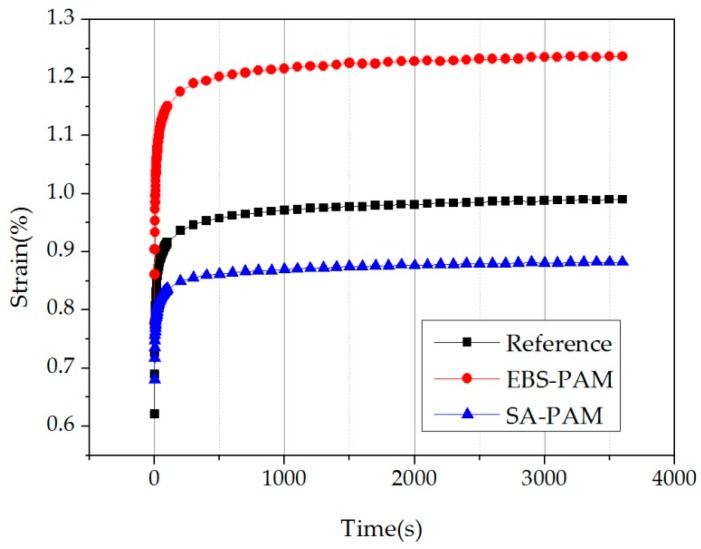
The creep curves of the three mixtures.

It can be seen from Figure 9 that the creep curves of the three mixtures are clearly divided into a migration period and a stabilization period. In the stabilization period, the creep deformation of EBS-PAM was significantly greater than that of the control group. In contrast, the creep deformation of SA-PAM was obviously smaller than that of the control group.

Furthermore, since the creep deformation of the three PAMs studied in this paper has only two phases, the classical viscoelastic model can be used to explicitly describe the creep behavior through the parameters of the model. Therefore, in order to further analyze the effect of the addition of two warming agents on the viscoelasticity of the PAM, the Burgers model was used. It is known from previous study [39] that the creep strain function under the Burgers model is as shown in Equation (4):

(4) $$\varepsilon (t)=\sigma_{0}[\frac{1}{E_{1}}+\frac{t}{\eta_{1}}+\frac{1}{E_{2}}(1-e^{\frac{-E_{2}t}{\eta_{2}}})]$$

where *E*_1_ is a coefficient that characterizes the instantaneous elastic deformation properties of the mixture during creep. The larger the *E*_1_, the smaller the instantaneous deformation of the mixture, and the deformation of the part can be completely recovered after unloading; *η*_1_ is a coefficient that characterizes the viscosity during creep. The larger the *η*_1_, the smaller the deformation of the mixture; the deformation of the part is permanent deformation and cannot be recovered after unloading. *τ* is the ratio of *E*_2_ and *η*_2_, which is an important time parameter for characterizing the viscoelastic properties of asphalt mixture. The larger the τ, the more the asphalt mixture shows viscosity, and the slower the recovery of deformation after unloading. According to the data obtained from the creep experiment, the fitting was performed with the Origin 9.0 software, and the four parameters obtained are shown in Table 10.

It can be seen in Table 10 that the fitting coefficient R^2^ of the Burgers model is not less than 0.94, indicating that the model can well describe the PAM in this experiment. Furthermore, the *E*_1_ values of EBS-PAM and SA-PAM were reduced by 20.7% and 6.3% relative to the control group, respectively. This indicates that the addition of EBS and SA increased the instantaneous elastic deformation of the PAM slightly, and reduced the ability of the mixture to resist transient deformation. For the *η*_1_ parameter, EBS-PAM was reduced by 13.8% relative to the control group, indicating that the permanent deformation increased during the creep process compared to the control group. To the opposite, SA-PAM increased by 67.8% relative to the control group, indicating that the permanent deformation of SA-PAM was reduced relative to the control group during creep. In addition, for the parameter *τ*, EBS-PAM and SA-PAM increased slightly relative to the control group, indicating a slight increase in the deformation recovery time.

In general, the permanent deformation of EBS-PAM during creep is greater than that of the control group, while the permanent deformation of SA-PAM is smaller than that of the control group, which shows that the application of SA could decrease the high-temperature permanent deformation of the mixture, thereby improving its high-temperature stability and enhancing its anti-rutting ability. This is also consistent with the results of the rutting test.

### 3.9. Resilient Modulus

The test results are shown in Table 11, which shows that the resilient modulus of EBS-PAM increased by 3.8% and the resilient modulus of SA-PAM decreased by 1.4% compared with that of the control group. It can be inferred that the addition of two kinds of warm-mixing agents has no significant effect on the resilient modulus of the PAM; that is, it will not significantly affect the performance of the PAM against traffic loading. 

## 4. Conclusions

In this paper, a warm mixed porous asphalt mixture with steel slag as aggregate and crumb-rubber–SBS-modified bitumen as a binder are designed and researched. Firstly, the Brookfield viscosity of three kinds of modified bitumen was tested. Based on the results, the mixing temperature of the warm-mixed PAM was determined. Then, the properties of the mixture were analyzed by mechanical experiments, and the effects of the addition of warming agents on the performance of the PAMs were also studied. From the test results, the following conclusions can be drawn:

(1) The addition of the warming additives significantly reduces the viscosity of the crumb-rubber–SBS-modified bitumen, which makes the mixing temperature of the porous asphalt mixture reduce by 10 °C compared to conventional mixing, which can reduce the construction difficulty of porous asphalt mixtures;

(2) The water stability and the Cantabro particle loss of the warm mix porous asphalt mixtures in this study meet the technical requirements of Chinese specifications. The Marshall stability and permeability of the warm-mix PAMs are better than that of the traditional hot-mix PAM;

(3) Based on the results of the rutting test, the uniaxial compression static creep test and the parameters of the Burgers model, the application of SA can greatly decrease the high-temperature permanent deformation of the porous asphalt mixture, thereby improving the anti-rutting ability of the mixture;

(4) The resilient moduli of the porous asphalt mixtures made at reduced temperatures did not change significantly, indicating that the application of the warming additives did not significantly alter the dynamic response of the porous asphalt mixture;

(5) Since the application of SA significantly improves the low-temperature crack resistance and high-temperature stability of the porous asphalt mixture, it is recommended to use SA as a warming additive in seasonal frozen regions, and the SA-PAM could be applied to sidewalks, parking lots and light traffic pavements in seasonal frozen areas.

## Figures and Tables

**Table 1:** Properties of SBS-modified bitumen.

Properties	Results	Chinese Standard
Penetration (25 °C, 0.1 mm)	65.2	60–80
Softening point (°C)	64.2	≥55
Ductility (5 °C, cm)	34.5	≥30
Flash point (°C)	264	≥230
Elastic recovery (25 °C, %)	91.7	≥65
After TFOT		
Mass loss (%)	0.34	≤±1.0
Penetration ratio (25 °C, %)	62	≥60
Ductility (5 °C, cm)	27	≥20

**Table 2:** Properties of crumb-rubber.

Properties	Results	Technical Criterion
Apparent density (g/cm^3^)	1.18	1.1–1.3
Metal content (%)	0.038	<0.05
Moisture content (%)	0.32	<1
Fiber content (%)	0.43	<1
Ash content (%)	4.5	≤8

**Table 3:** Properties of steel slag.

Properties	Results	Chinese Standard	Specification
Apparent specific density (g/cm^3^)	3.527	≥2.6	T 0304
Los Angeles abrasion (%)	12.9	≤28	T 0317
Flakiness content (%)	4.56	≤10	T 0312
Crushed stone value (%)	13.9	≤26	T 0316

**Table 4:** Void characteristics and permeability coefficient. PAM: porous asphalt mixture.

Types of Mixture	Voids in PAMs (%)		Permeability Coefficient	
	Mean	Sd	Mean	Sd
Control group	21.5	0.3	0.3	0.012
EBS-PAM	23.2	1.4	0.33	0.014
SA-PAM	23.8	1.3	0.34	0.012
Chinese standard	≥18		≥0.28	

**Table 5:** Marshall stability and flow value.

Types of Mixture	Stability (KN)		Flow Value (mm)	
	Mean	Sd	Mean	Sd
Control group	8.20	0.8	2.25	0.03
EBS-PAM	8.29	0.6	2.76	0.02
SA-PAM	9.71	0.4	2.37	0.04
Chinese standard	≥5.0		2–4	

**Table 6:** Marshall stability comparison.

Types of Mixture	Aggregate	Voids in Mixture (%)	Stability (KN)	Flow Value (mm)
PAM	Steel Slag	21.5	8.20	2.25
PAM	Basalt	20.3	6.21	2.9

**Table 7:** The results of the freeze–thaw splitting test.

Types of Mixture	Splitting Strength (MPa)		Freeze–Thaw Splitting Strength (MPa)		Freeze–Thaw Splitting Strength Ratio (%)
	Mean	Sd	Mean	Sd	
Control group	0.628	0.026	0.588	0.012	93
EBS-PAM	0.628	0.030	0.543	0.020	86
SA-PAM	0.629	0.012	0.552	0.016	87
Chinese standard	-		-		≥85

**Table 8:** Dynamic stability.

Types of Mixture	*d*_1_ (mm)	*d*_2_ (mm)	Dynamic Stability (wheel pass/mm)
Control group	2.14	2.29	4200
EBS-PAM	2.81	3.18	1703
SA-PAM	1.98	2.10	5250

**Table 9:** Cantabro particle loss.

Types of Mixture	Cantabro Particle Loss (%)		Chinese Standard
	Mean	Sd	
Control group	9.5	0.21	≤15
EBS-PAM	13.8	0.40	≤15
SA-PAM	12.3	0.32	≤15

**Table 10:** The fitting results.

Types of Mixture	*E*_1_	*η*_1_	*E*_2_	*η*_2_	*τ*	*R* ^2^
Control group	58.3	1.37 × 10^6^	185.5	3064	16.5	0.94
EBS-PAM	46.2	1.18 × 10^6^	142	2488	17.5	0.94
SA-PAM	54.6	2.30 × 10^6^	358.5	7193.4	20.0	0.94

**Table 11:** Results of resilient modulus.

Types of Mixture	Pressure/KN	Horizontal Stress /KPa	Horizontal Deformation	Resilient Modulus
Control group	1.6	169.6	5.0	3396
EBS-PAM	1.7	180.6	5.1	3527
SA-PAM	1.7	162.7	5.0	3347
